# Is Timing of Steroid Exposure Prior to Immune Checkpoint Inhibitor Initiation Associated with Treatment Outcomes in Melanoma? A Population-Based Study

**DOI:** 10.3390/cancers14051296

**Published:** 2022-03-02

**Authors:** Nikita Nikita, Joshua Banks, Scott W. Keith, Andrew Song, Jennifer M. Johnson, Melissa Wilson, Swapnil Sharma, Grace Lu-Yao

**Affiliations:** 1 Department of Medical Oncology, Thomas Jefferson University, Philadelphia, PA 19107, USA; fnu.nikita@jefferson.edu (N.N.); jennifer.m.johnson@jefferson.edu (J.M.J.); melissa.wilson@sluhn.org (M.W.); swapnil.sharma@jefferson.edu (S.S.); 2 Department of Medical Oncology, Sidney Kimmel Cancer Center (SKCC) at Jefferson, Philadelphia, PA 19107, USA; 3 Department of Pharmacology & Experimental Therapeutics, Division of Biostatistics, Thomas Jefferson University, Philadelphia, PA 19107, USA; joshua.banks@jefferson.edu (J.B.); scott.keith@jefferson.edu (S.W.K.); 4 Department of Radiation Oncology, Thomas Jefferson University, Philadelphia, PA 19107, USA; song.andrew.j@gmail.com; 5 College of Population Health, Thomas Jefferson University, Philadelphia, PA 19107, USA

**Keywords:** immunotherapy, steroids, melanoma, overall mortality, all-cause mortality, hospitalizations, immune checkpoint inhibitors

## Abstract

**Simple Summary:**

Immune checkpoint inhibitors (ICIs) have revolutionized the cancer treatment landscape, yet the impact of the timing of the use of an immunosuppressive agent such as steroids on ICI outcomes is poorly described. Utilizing the SEER-Medicare-linked dataset, this study explored the relationship between timing of steroid exposure preceding ICI administration and subsequent treatment outcomes in melanoma. We found steroid use up to 3 months prior increased risk for mortality up to 6 months after ICI initiation. Furthermore, steroid use was a significant contributor to poorer outcomes from ICIs, which should be considered before prescribing ICIs.

**Abstract:**

Immune checkpoint inhibitors (ICIs) harness the immune system and are the therapy of choice for multiple cancers. Although immunosuppressive agents such as steroids are also used in many cancers, it is unknown how their timing affects treatment outcomes. Thus, we investigated the relationship between the timing of steroid exposure preceding ICI administration and subsequent treatment outcomes in melanoma. This population-based study utilized the SEER-Medicare-linked database to identify patients diagnosed with melanoma between 1991 and 2015 and receiving ICIs between 2010 and 2016, examining last steroid exposure in the 12 months preceding ICI. The main outcome was all-cause mortality (ACM) after ICIs. Modifications of the Cox proportional hazards model were used to calculate time-dependent hazards. Of 1671 patients with melanoma receiving ICIs, 907 received steroids. Compared with no steroids, last steroid exposures ≤1 month and 1–3 months prior to ICIs were associated with a 126% and 51% higher ACM within 3 months post ICI initiation, respectively (hazard ratio (HR): 2.26, 95% CI: 1.65–3.08; and HR: 1.51, 95% CI: 1.01–2.27). Steroid exposure within 3 months of initiating ICIs was associated with increased mortality up to 6 months after ICI. Further investigation is warranted to elucidate mechanisms affecting outcomes due to steroids.

## 1. Introduction

According to the National Cancer Institute, approximately 40% of individuals in the United States (US) will be diagnosed with cancer in their lifetime [1]. Since first being approved in 2011 by the US Food and Drug Administration, immune checkpoint inhibitors (ICIs) such as ipilimumab (cytotoxic T-lymphocyte-associated antigen 4 (CTLA-4) inhibitor), pembrolizumab, and nivolumab (programmed cell death protein (PD-1) inhibitors) have played a significant role in survival after cancer diagnosis. ICIs, which harness the immune system and amplify its natural ability to fight cancer, have produced improved outcomes and increased survival rates, thereby becoming a major pillar of cancer therapy. Providing significant improvements in survival, durable responses, and comparatively fewer adverse events, ICIs are currently used in multiple cancers, such as unresectable hepatocellular carcinoma, non-small cell lung cancer, renal cell carcinoma, colorectal cancer, etc. [2,3,4,5]. In cutaneous melanoma, they are used in Stage III/IV resectable disease as adjuvant therapy, in Stage IV unresectable melanoma as primary therapy, and in recurrent disease [6,7,8,9].

Melanoma was the first site to be approved for ICIs; since then, ICIs have measurably improved the prognosis of metastatic melanoma by reversing effector T-cell dysfunction, thereby strengthening anti-tumor immune response [10,11]. A recent update of the Phase-III CheckMate 067 trial for melanoma found that ipilimumab and nivolumab combination has the highest five-year survival for melanoma (52%) compared to nivolumab alone (44%) and ipilimumab alone (26%) [3]. Despite the efficacy of ICIs, this improvement in survival is limited only to 30–40% of patients [10,12,13]; hence, it is important to identify factors that drive ICI response to optimize treatment strategies.

One in five metastatic melanoma patients has brain metastasis at the time of diagnosis, and three of four patients develop it over time. Traditionally, these are treated with systemic steroids. Steroid use impairs the immune system at various levels, from inhibition of acute inflammation to immunomodulatory effects [14]. This raises a major concern since ICIs require a robust immune response. Moreover, steroids are widely used in cancer patients and are known to have a far-reaching impact, ranging from curative treatment in certain cancers such as leukemia and lymphoma to supportive therapy in brain metastases [15,16,17,18,19,20].

Most clinical trials of ICIs have excluded patients treated with immunosuppressive agents (such as prednisone ≥10 mg) that can impair T-cell functions [21,22,23,24]. Therefore, data are limited on the outcomes for patients not eligible for ICI trials and receiving ICI in the real world. Several institution-based studies, primarily in advanced/metastatic non-small cell lung cancer (NSCLC), have shown worsened clinical outcomes with steroid exposure at baseline [25] or after initiation of ICI [26], including decreased overall response rate, shorter progression-free survival, and lower overall survival. It has also been reported that the use of steroids for immune-related adverse events concurrently with ICIs does not impact survival among patients [5]. Therefore, the impact of steroid use on the effectiveness of ICIs is debatable.

Specifically, the relationship between the timing of steroid exposure prior to ICI initiation and subsequent outcomes for patients with melanoma remains unknown. It is also unclear how long the effects of steroids may last. Thus, we conducted a population-based study to fill these knowledge gaps by focusing on melanoma patients using steroids prior to ICI initiation and their treatment outcomes.

## 2. Materials and Methods

This study was approved by the Institutional Review Board at Thomas Jefferson University IRB 17D.389. 

### 2.1. Data Source

We utilized the Surveillance, Epidemiology, and End Results linked Medicare (SEER-Medicare) database for this study, which includes all patients with cancer aged 65 and over. As of 2017, the SEER program covers approximately 31% of the US population with a 98% case ascertainment rate, and it collects data on cancer status at diagnosis, primary cancer therapy, demographic characteristics, and cause of death among patients with cancer [27]. Medicare is the major health insurance provider among Americans aged 65 years and over and covers approximately 18% of the entire US population [28]. Part A of Medicare provides coverage for hospital care, skilled nursing facility services, other institutional care, some home health care, and hospice care [29]. Part B covers services from physicians, other health professionals, ambulatory surgical centers, outpatient dialysis, home health, and other ambulatory services [29]. Part D covers prescription drugs under private drug plans [29].

### 2.2. Study Participants

We identified patients diagnosed with melanoma between 1 January 1991 and 31 December 2015 from the patient entitlement and diagnosis summary files. For patients with multiple diagnoses of melanoma, the most recent melanoma diagnosis was used to identify patients. Patients were excluded if melanoma was diagnosed at death or autopsy. Patients were also excluded if they were enrolled in a health maintenance organization, or had no Medicare parts A, B, or D coverage during the study period.

### 2.3. Drug Exposures

The ICIs included in the study were ipilimumab (YERVOY^®^; identified using the Healthcare Common Procedure Coding System (HCPCS) code “J9228”), nivolumab (OPDIVO^®^; HCPCS code “J9229”), pembrolizumab (KEYTRUDA^®^; HCPCS code “J9271”), and ipilimumab/nivolumab combination. The last prescription of all intravenous and injectable steroids prescribed within 12 months prior to ICI initiation were included in the study. For oral steroids, the last date of steroid exposure was calculated by adding the information on days of supply to the last prescription date. All inhalational and topical steroids were excluded from the analysis. In the database, medication follow-up information was available until 31 December 2016 for all patients. Therefore, all drug exposures were identified from Medicare claims between 1 January 2010 and 31 December 2016.

### 2.4. Steroid-Exposure Timing

According to the timing of their last steroid exposure during the 12 months prior to ICI initiation, patients were grouped into one of four categories: (a) ≤1 month prior, (b) 1 month to ≤3 months prior, (c) 3 to 12 months prior, or (d) no steroids (reference group).

### 2.5. Endpoints

The primary endpoint was all-cause mortality (ACM). The survival time was defined as the time from the first dose of ICIs until death or the end of the follow-up period. The secondary endpoint was the risk of inpatient ER and non-ER hospitalization following the initiation of ICIs. Patients were followed until death or the last follow-up date, 31 December 2016.

### 2.6. Descriptive Variables

Demographic variables included in the study were age at the time of diagnosis, sex, race, marital status, income, SEER region, year of diagnosis, and state buy-in. Clinical variables included in the study were cancer stage at the time of diagnosis and CCI. 

### 2.7. Statistical Analysis

Descriptive statistics were presented for continuous variables as medians with interquartile ranges, and for categorical variables as frequency counts with percentages and chi-square tests.

### 2.8. Modeling All-Cause Mortality (ACM)

Median survival was estimated using Kaplan–Meier methods compared by log-rank test [30]. The crude overall ACM incidence rates were estimated as the number of deaths divided by the number of patients at risk by steroid-exposure timing groups. Cox modeling was used to examine the association between ACM and the timing of the last steroid exposure after adjusting for all demographic and clinical variables [31]. The Schoenfeld residual plots were assessed for time dependence between last steroid-exposure timing prior to ICI initiation and ACM. We observed time-dependent hazards that violated the proportional hazards assumption. To address this, we programmed survival time indicator variables to interact with the steroid-timing-exposure indicator variables in the model to represent the interaction between steroid-exposure timing and follow-up time periods, allowing the hazards model coefficients to change during survival at 3 months and again at 6 months after initiating ICI [32].

### 2.9. Modeling Hospitalizations

We stratified hospitalizations as ER or non-ER because ER hospitalizations are associated with more severe symptoms and higher cost to the patient as well as the health system [33]. The counting process approach was used for structuring the survival data for recurrent events as well as for computing the time to hospitalization events for non-ER and ER hospitalizations, respectively [34]. This method is an extension of the Cox modeling framework, which is useful when an outcome event of interest can recur. The time at risk for hospitalization for each patient was calculated as the difference between the time of ICI initiation and the time of death or the end of the follow-up period. These models were used to estimate hospitalization HRs adjusted for covariates, including age, sex, Charlson comorbidity index (CCI), marital status, and the year of diagnosis for each patient. The proportional hazards assumption was violated, so we applied the same approach to allow the hospitalization–hazards model coefficients to change during survival at 3 months and again at 6 months, after initiating ICI as described above for modeling ACM.

### 2.10. Pre-Planned Evaluation of Unmeasured Confounding Effects

To assess the robustness of our study results, we used E-values to evaluate the potential effects that unmeasured confounding would need to have in order to change the conclusions [35]. The E-value measures the minimum strength of association that an unmeasured confounder must have with both the exposure and outcome, while taking into account all potential covariates, to negate the observed exposure–outcome association by making the estimated HR equal to 1.

The significance level of all tests was set a priori at the 0.05 level. We performed all the analyses with SAS version 9.4 (SAS Institute Inc., Cary, NC, USA).

## 3. Results

**Figure 1:**
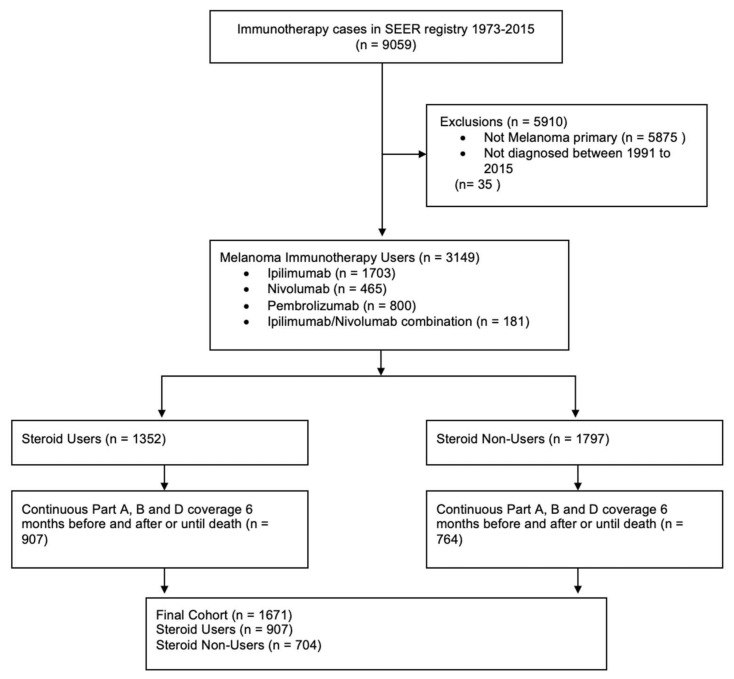
Consort flow diagram.

We identified 1671 eligible patients for this study (858 prescribed ipilimumab, 258 prescribed nivolumab, 456 prescribed pembrolizumab, and 99 prescribed an ipilimumab/nivolumab combination) (Figure 1). Of these, 864 (51.70%) were exposed to steroids within 12 months prior to ICI initiation. Using the sequence variable, we identified that for 89.7% of the patients, melanoma was either the only cancer diagnosis in the dataset or was the first cancer to be diagnosed. The numbers of patients exposed to steroids within 1 month, 1 to ≤3 months, and 3 to 12 months prior to ICI initiation were 343, 215, and 306, respectively. The demographics and clinical characteristics of patients exposed and those not exposed to steroids were similar (Table 1). Patients (median age 68) were predominantly male (71.2%), white (97.3%), married (61.5%), or non-metastatic (M0) at the time of primary diagnosis (77.3%) (Table 1). The median survival for patients without steroid exposure was 20.5 months and median survival for patients without steroid exposure was 20.5 months, median survival for those exposed to steroids 4–12 months prior was 21.2 months, median survival for those exposed 1 to ≤3 months prior was 14.0 months, and median survival for those exposed ≤1 month prior was 8.9 months (*p* < 0.05; Table S1).

### 3.1. ACM and Steroid Exposure Prior to ICI Initiation

**Figure 2:**
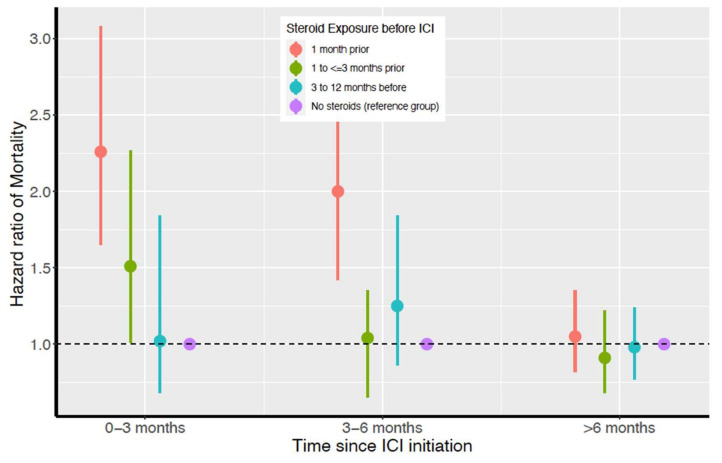
Time-dependent all-cause mortality hazard ratios for all steroid-exposure groups and survival time periods.

Figure 2 and Table 2 depict the results from the time-dependent hazards model for ACM, evaluating its association with steroids prior to ICIs. Exposures to steroids ≤1 month and 1 to ≤3 months prior to ICI initiation were associated with a 126% and 51% higher ACM within 3 months post ICI initiation, respectively (HR = 2.26; 95% CI: 1.65–3.08; *p* < 0.001 for 1 month prior and HR = 1.51; 95% CI: 1.01–2.27; *p* = 0.04 for 1 to ≤3 months prior). Similarly, steroid exposure ≤1 month prior to ICI initiation was associated with a 100% increase in ACM 3 to 6 months after ICI initiation (HR = 2.00; 95%CI: 1.42–2.82; *p* < 0.001). In general, the nearer the last steroid exposure to ICI initiation, the higher the ACM risk. This association followed an approximately linear trend with a statistically significant non-zero slope (*p* < 0.001).

### 3.2. ER-Hospitalization Risk and Timing of Steroid Exposure before ICI initiation

**Figure 3:**
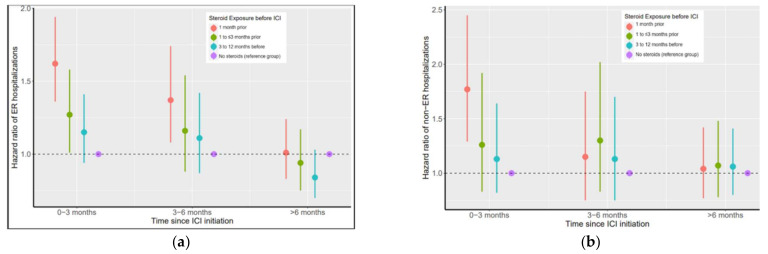
Time-dependent ER (panel a) and non-ER hospitalization (panel b) hazard ratios for all steroid-exposure groups and survival time periods. (**a**) Steroid exposure prior to ICI use is associated with higher ER hospitalization rates up to 6 months post ICI initiation. Steroid exposure up to 3 months prior is associated with higher ER hospitalization rates up to 6 months post ICI initiation. The elevated risk of ER hospitalization associated with prior steroid exposure diminished over time and vanished by 6 months post ICI. (**b**) For non-ER hospitalizations, steroid exposure 1 month prior is associated with higher non-ER hospitalizations up to 3 months post ICI initiation and steroid exposure 1–3 months prior is associated with higher non-ER hospitalization rates up to 6 months post ICI initiation. The elevated risk of non-ER hospitalization associated with prior steroid exposure diminished over time and vanished by 6 months post ICI.

Steroid exposure within 12 months of ICI initiation was also associated with a higher incidence of ER hospitalizations. Figure 3 panel A and Table 3 show the HRs and 95% CI for ER hospitalizations post ICI initiation for different steroid-exposure timing groups. Steroids within 1 month prior to ICI initiation was associated with a 62% higher incidence of ER hospitalizations during the first 3 months (HR = 1.62; 95% CI: 1.36–1.94; *p* < 0.001) and a 37% higher incidence of ER hospitalizations during the next 3 months of survival post ICI initiation (HR: 1.36; 95% CI: 1.08–1.74; *p* = 0.01). Steroid exposure within 1 to ≤3 months prior was associated with a 27% higher incidence of ER hospitalization during the first 3 months post initiation (HR: 1.27; 95% CI: 1.01–1.58; *p* = 0.037). Similarly, steroid exposure 3 to 12 months prior was associated with higher HR for ER hospitalizations 3 months post ICI initiation compared to patients with no prior steroids (Table 3). Beyond the first six months post ICI initiation, steroid exposure was not associated with an increase in ER hospitalizations.

### 3.3. Non-ER-Hospitalization Risk and Steroid Exposure before ICI Initiation

Figure 3 panel B and Table S2 show the HRs and 95% CI for non-ER hospitalizations post ICI initiation for different steroid-exposure timing groups. For the first 3 months of survival post ICI-initiation, steroid exposure within ≤1 month prior was associated with a 77% higher risk of non-ER hospitalizations (HR = 1.77; 95% CI: 1.29–2.45; *p* = 0.005).

### 3.4. Pre-Planned Analysis to Evaluate Unmeasured Confounding

The E-values in Table 4 suggest the minimum strength of unmeasured confounding association that would nullify the HRs to which they specifically correspond in Figure 2. It is unlikely that an unmeasured or unknown confounder would change our HR results for ACM during the first 6 months of survival after ICI initiation associated with steroid exposure within 1 month prior to ICI initiation. The confounder would need to have a relative risk association of at least 3.27 with both ACM and exposure to steroids within 1 month prior to ICI initiation. It would need to have relative risk associations greater than 3.44 for both ACM and steroid exposure 1 month prior.

## 4. Discussion

This is the first population-based study to demonstrate a strong association between the timing of steroids preceding ICI initiation and clinical outcomes among patients with melanoma. We found that shorter time between last steroid exposure and ICI initiation was associated with significantly worse outcomes. Specifically, steroid exposure within 3 months of initiating ICIs was associated with increased mortality during the first 6 months after ICI initiation, with risk elevated at least twofold if steroids were used within 1 month prior to initiation. Additionally, these patients were more likely to have ER and non-ER hospitalizations when exposed to steroids within 1 month prior to ICI initiation. These findings are thought-provoking given the concerns for the potential ICI-suppressive effects of steroids. Our data suggest potential interplay between ICIs and immunosuppressive agents; therefore, oncologists and cancer caregivers are advised to take into account the potential effects of immunosuppressive agents such as steroids before initiating ICIs.

Our findings are consistent with another retrospective study that found that baseline steroid exposure within 30 days of receiving ICIs for NSCLC was associated with worse ACM after adjusting for covariates, including history of brain metastases [25]. Another study using the large Flatiron dataset found that baseline steroid exposure was associated with worse ACM among patients with NSCLC and melanoma [36]. We found a similar trend in our study; patients with prior steroid exposure had worse ACM compared to those without steroid exposure. Moreover, we suspect that the ACM in our study population could be higher compared to results from clinical trials, since clinical trials usually exclude patients with active, untreated metastases, and those with high baseline or chronic steroid exposure. In our study, patients exposed to steroids within 1 month before ICI initiation did much worse than the expected mortality; median OS was 8.9 months for patients with steroids exposure within 1 month prior to ICI compared to 11.4 months in a meta-analysis of over 1600 patients [20]. We posit that the increase in short-term ACM is suggestive of an acute medical process. However, the exact mechanism is unknown and further studies are needed.

Exposure to steroids prior to ICI initiation was associated with higher risk of ER hospitalizations post ICI. Due to data limitations, we were unable to address whether the increased hospitalizations were a result of immune-related adverse events or steroid exposure. To minimize unmeasured confounding effects, we chose to model risk of hospitalization (6 months post ICI vs. 6 months before ICI) because socioeconomic status, access to care, health habits, and comorbidities were likely to have modest changes within a short period of time. In addition, we used E-values to assess the robustness of the findings and found that any unmeasured confounding would require a high HR to nullify the major conclusions of the study.

Melanoma was the first cancer approved for ICIs and thus has the most mature data for analyzing steroids preceding ICI initiation. Unlike NSCLC, where COPD and brain metastases are more likely to require steroids [37], the indications for steroids preceding ICIs in our population is unclear, since the SEER-Medicare database does not reliably capture metastasis information beyond initial cancer diagnosis. However, having at least one comorbidity increased the likelihood of steroid exposure, which could signal poorer health overall. Although patients with CCI ≥ 2 had higher mortality risk and hospitalization risks in all cohorts, steroid exposure remained an independently prognostic factor after adjusting for comorbidity and relevant covariates. Thus, our findings are robust among melanoma patients with steroid exposure.

### 4.1. Potential Mechanisms

One potential explanation for these results is the role played by T cells in the mechanism of action for steroids and ICIs. Steroid exposure is associated with suppression of T-cell activity, leading to impaired immune function and inferior outcomes [38], whereas ICIs require the activation of T cells for antitumor activity. Therefore, steroid exposure prior to ICIs may result in decreasing the efficacy of ICIs due to suppression of the CD8-positive T cells that are required for an ICI blockade [24]. A preclinical study found that dexamethasone increased PD-1 expression in certain subsets of T cells, therefore suggesting that inhibition of PD-1 might overcome T-cell exhaustion [39]. Another study found that dexamethasone use blocked the proliferation of T cells and attenuated the stimulation of CD28 cells more than memory T cells. A mouse model further found that although CTLA-4 inhibition partially restored tumor infiltration of T cells alongside consequent dexamethasone exposure, there was no significant increase in CD8 T cells, hence suggesting that early exposure to steroids led to significantly suppressed ICI response. Given these findings, we propose that patients using steroids at baseline should be included in future clinical trials and data analysis in order to optimize results. To this effect, the NCI is currently working with multiple cancer sites to conduct two randomized clinical trials among patients with autoimmune diseases (most using steroids) and cancer receiving nivolumab: NCT03656627 (Nivolumab in Treating Patients with Autoimmune Disorders or Advanced, Metastatic, or Unresectable Cancer) and NCT03816345 (A Phase Ib Study of Nivolumab in Patients With Autoimmune Disorders and Advanced Malignancies (AIM-NIVO)) [40,41]. Until further information regarding the safety of such concurrent medication use is available, ICIs must be administered with caution and after careful assessment of the risk–benefit ratio for each patient.

### 4.2. Strengths and Limitations

The study has several strengths. First, derived from a large, population-based database with broad representation, our study offers excellent real-world generalizability. Second, this is the first study to address the timing effects associated with steroid exposure within 12 months prior to ICI initiation. Previous studies have not examined the timing effects beyond 3 months prior. Third, this is the first study using national-level data specific to Medicare patients receiving ICIs. Fourth, this is the first study to include differences in hospitalization risks stratified by ER and non-ER hospitalization among patients exposed to steroids prior to receiving ICIs. Since ER hospitalizations are associated with higher cost to the patients as well as the health system, it is important to understand the difference between the association of steroid exposure and ICIs for both ER and non-ER hospitalizations.

The study also has limitations. First, given the retrospective nature of the study, we were unable to formulate a cause–effect relationship between the timing of steroid exposure and the clinical outcomes. Second, the SEER dataset does not provide information about cancer staging beyond the date of diagnosis; however, based on the treatment dates in our cohort, we assume that all patients receiving ICIs during the study period had advanced-stage disease since ICIs were only approved for advanced stage disease during this time window. Third, we did not have any data on laboratory values such as PD-1 status, neutrophil count, neutrophil-to-lymphocyte ratio, tumor mutation burden, or line of therapy, which limited our analysis. Fourth, due to the limitation of the dataset, we were unable to identify the dosage of steroids. Fifth, we defined steroid exposure using only prescription data, not whether or for how long the steroids were actually used. Thus, there may be some misclassification of steroid exposure. Further studies are warranted to confirm the relationship between steroid exposure and clinical outcomes of ICIs. These studies should focus on the dosing of steroids, the type of steroid used, and the difference between acute and chronic steroid exposure.

## 5. Conclusions

The nearer the last steroid exposure to ICI initiation, the higher the ACM risk; thus, care providers are advised to take this into account and consider the risk–benefit ratio when making therapeutic decisions. It is recommended that the care team consider delaying steroid exposure and focus on steroid-sparing strategies before initiating ICIs or consider delaying initiating ICIs for 3 months to allow for steroid washout.

## Figures and Tables

**Table 1:** Demographic and clinical characteristics of the study sample by steroid-exposure status in the 12 months prior to ICI initiation.

Characteristic	Total Participants (*N =* 1671)	No Steroids (*N* = 807)	Steroids (*N* = 864)	*p*-Value
Sex				0.088
Male	1189 (71.2)	590 (73.1)	599 (69.2)	
Female	482 (28.9)	217 (26.9)	265 (30.7)	
Race				0.702
White	1626 (97.3)	784 (97.2)	842 (97.5)	
Non-white	45 (2.7)	23 (2.9)	22 (2.5)	
Marital status				0.234
Missing and unknown	193 (11.6)	86 (10.7)	107 (12.4)	
Single (never married), Unmarried or domestic partner (same sex or opposite sex or unregistered)	196 (11.7)	97 (12.0)	99 (11.5)	
Widowed, divorced, and separated	254 (15.2)	136 (16.9)	118 (13.7)	
Married (including common law)	1028 (61.5)	488 (60.5)	540 (62.5)	
Age at diagnosis, median (IQR)	69 (63–75)	69 (64–76)	68 (63–74)	
Age at diagnosis				0.009
<60	244 (14.6)	113 (14.0)	131 (15.2)	
60–69	648 (38.8)	295 (36.6)	353 (40.9)	
70–79	583 (32.9)	283 (35.1)	300 (34.7)	
80+	196 (11.7)	116 (14.4)	80 (9.3)	
Age at first ICI use, median (IQR)	75 (70–81)	75 (70–81)	74 (69–80)	
Year of diagnosis				0.554
1991–1999	107 (6.4)	46 (6.0)	61 (6.7)	
2000–2005	278 (16.6)	129 (16.9)	149 (16.4)	
2006–2010	470 (28.1)	204 (26.7)	266 (29.3)	
2011–2015	816 (48.8)	385 (50.4)	431 (47.5)	
Sequence				0.094
Only	807 (48.3)	411 (50.9)	396 (45.8)	
1st	691 (41.4)	313 (38.8)	378 (43.8)	
Subsequent (2nd–11th)	173 (10.4)	83 (10.3)	90 (10.4)	
Charlson comorbidity index				**0.043**
0	973 (58.2)	495 (61.4)	478 (55.4)	
1	336 (20.1)	152 (18.9)	184 (21.3)	
≥2	360 (21.5)	159 (19.7)	201 (23.2)	
SEER region				0.185
Northeast	386 (23.1)	196 (24.3)	190 (21.6)	
South	344 (20.6)	150 (18.6)	194 (22.5)	
North Central	132 (7.9)	69 (8.6)	63 (7.3)	
West	809 (49.4)	392 (48.6)	417 (48.3)	
State buy in ^1^				0.080
Yes	255(15.2)	136 (16.9)	119 (13.8)	
No	1416 (84.7)	671 (83.2)	745 (86.2)	
Clinical T stage at diagnosis				**0.01**
T0	91 (5.5)	32 (4.0)	59 (6.8)	
T1	322 (19.3)	165 (20.5)	157 (18.2)	
T2	178 (10.7)	79 (9.8)	99 (11.5)	
T3	70 (4.2)	36 (4.5)	34 (3.9)	
T4	706 (42.3)	362 (44.9)	344 (49.1)	
TX	284 (17.0)	128 (15.9)	156 (18.1)	
Clinical N stage at diagnosis				0.675
N0	964 (57.7)	459 (55.9)	505 (58.5)	
N1	210 (12.6)	109 (13.5)	101 (11.7)	
NX	119 (7.12)	59 (7.3)	60 (6.9)	
missing	377 (22.6)	180 (22.3)	197 (22.8)	
Clinical M stage at diagnosis				0.432
M0	1292 (77.3)	631 (78.2)	661 (76.5)	
All M1	153 (0.1)	65 (0.1)	88 (0.1)	
MX	194 (11.6)	91 (11.3)	103 (11.9)	
Missing	32 (1.9)	20 (2.5)	12 (1.4)	
Melanoma specific mortality as of 31 December 2016				0.712
Dead	398 (23.8)	189 (22.4)	209 (24.2)	
Alive	1273 (76.2)	618 (76.6)	655 (75.8)	
All-cause mortality as of 31 December 2016				**0.011**
Dead	1031 (61.7)	482 (59.7)	549 (63.5)	
Alive	640 (38.3)	325 (40.3)	315 (36.5)	

^1^ State buy-in: indicating that the state pays part or all of the patient’s Medicare Part B premium or that the person is in the Medicaid program, bolt values represent significant values.

**Table 2:** Steroid-exposure timing prior to ICI initiation and its time-dependent association with all-cause mortality after ICI initiation.

Timing of Steroid Exposure Prior to ICI Initiation	0 to ≤3 Months Post ICI Initiation Hazard Ratios ^1^ (95% CI)	3 to ≤6 Months Post ICI Initiation Hazard Ratios (95% CI)	≥6 Months Post ICI Initiation Hazard Ratios (95% CI)
No steroids in 12 months before ICI	Ref	Ref	Ref
Steroids ≤ 1 month prior to ICI	2.26 (1.65–3.08) ^2^	2.00 (1.42–2.82) ^2^	1.05 (0.82–1.35)
Steroids 1 to ≤3 months prior to ICI	1.51 (1.01–2.27) ^2^	1.04 (0.65–1.35)	0.91 (0.68–1.22)
Steroids 3 to 12 months prior to ICI	1.02 (0.68–1.52)	1.25 (0.86–1.84)	0.98 (0.77–1.24)

^1^ Hazard ratios estimated by time-dependent hazards model adjusted for sex, age, marital status, sequence of cancer diagnosis, year of diagnosis, and Charlson comorbidity index. ^2^ *p* < 0.001.

**Table 3:** Steroid-exposure timing prior to ICI initiation and its time-dependent association with ER hospitalization after ICI initiation.

Timing of Steroid Exposure Prior to ICI Initiation	0 to ≤3 Months Post ICI Initiation Hazard Ratios ^1^ (95% CI)	3 to ≤6 Months Post ICI Initiation Hazard Ratios ^1^ (95% CI)	≥6 Months Post ICI Initiation Hazard Ratios ^1^ (95% CI)
No steroids in 12 months before ICI	Ref	Ref	Ref
Steroids ≤1 month prior to ICI	1.62 (1.36–1.94) ^2^	1.37 (1.08–1.74) ^2^	1.01 (0.83–1.24)
Steroids 1 to ≤3 months prior to ICI	1.27 (1.01–1.58) ^2^	1.16 (0.88–1.54)	0.94 (0.75–1.17)
Steroids 3 to 12 months prior to ICI	1.15 (0.94–1.41)	1.11 (0.87–1.42)	0.84 (0.70–1.03)

^1^ Hazard ratios estimated by time-dependent hazards model adjusted for sex, age, marital status, sequence of cancer diagnosis, year of diagnosis, and Charlson comorbidity index. ^2^ *p* < 0.001.

**Table 4:** Assessment of the robustness (using E-values ^1^) of observed associations for all-cause mortality with steroid exposure presented in Figure 2.

Timing of Steroid Exposure Prior to ICI Initiation	0 to ≤3 Months Post ICI Initiation Hazard Ratios	3 to ≤6 Months Post ICI Initiation Hazard Ratios	≥6 Months Post ICI initiation Hazard Ratios ^1^
Steroids ≤ 1 month prior to ICI	3.27	3.44	1.58
Steroids 1 to ≤ 3 months prior to ICI	2.82	1.77	1.49
Steroids 3 to 12 months prior to ICI	1.89	1.69	1.40

^1^ E-value measures the minimum strength of association that an unmeasured confounder must have with both the exposure and outcome, and considers the covariates to nullify the observed exposure–outcome association. The further an E-value shown here is from 1, the less likely that unmeasured confounding would account for its corresponding HR in Figure 2.

## Data Availability

The data reported in this study can be obtained by writing to G.L., the corresponding author.

## Supplementary Materials

The following are available online at https://www.mdpi.com/article/10.3390/cancers14051296/s1, Table S1: Median survival and cumulative incidence of all-cause mortality 6 months post ICI initiation, Table S2: Timing of steroid exposure prior to ICI initiation and its time-dependent association with non-ER hospitalization.
