# Supplementary material for: Is Timing of Steroid Exposure Prior to Immune Checkpoint Inhibitor Initiation Associated with Treatment Outcomes in Melanoma? A Population-Based Study
Source: Cancers (Basel). 2022 Mar 2;14(5):1296. doi: 10.3390/cancers14051296 (PMC8909505; doi:10.3390/cancers14051296)
Supplement: Supplementary file 1 [file cancers-14-01296-s001.zip › cancers-1547607-supplementary.pdf]

cancers MDPI

Supplementary Tables

Is timing of steroid exposure prior to immune checkpoint inhibitor initiation associated with treatment outcomes? A population-based study

Table S1. Median Survival and Cumulative Incidence of All-Cause Mortality 6-months post ICI initiation.

Timing of steroid exposure prior to ICI initiation	Median Survival (months)	Cumulative Incidence of ACM 6 months post ICI initiation; % (95% CI)
No Steroids in 12 months before ICI	20.47 (18.23–23.00)	23.87 (21.93–25.87)
Steroids ≤ 1 month prior to ICI	8.93 (6.97–10.67)	42.66 (38.65–46.61)
Steroids 1 to ≤3 months prior to ICI	14.00 (10.13–21.10)	34.43 (20.39–28.26)
Steroids 3 to 12 months prior to ICI	21.23 (18.47–27.47)	24.23 (20.39–28.26)

Table S2. Timing of steroids exposure prior to ICI initiation and its time-dependent association with non-ER hospitalization.*.

Timing of steroid exposure prior to ICI initiation	0 to ≤3 months post ICI initiation Hazard Ratio (95% CI)	3 to ≤6 months post ICI initiation Hazard Ratio (95% CI)	≥6 months post ICI initiation Hazard Ratio (95% CI)
No Steroids in 12 months before ICI	Ref	Ref	Ref
Steroids ≤ 1 month prior to ICI	1.77 (1.29–2.45)**	1.15 (0.75–1.75)	1.04 (0.77–1.42)
Steroids 1 to ≤3 months prior to ICI	1.26 (0.83–1.92)	1.30 (0.83–2.02)	01.07 (0.78–1.48)
Steroids 3 to 12 months prior to ICI	1.13 (0.82–1.64)	1.14 (0.75–1.70)	1.06 (0.80–1.41)

* Hazard ratios estimated by time-dependent recurrent event hazards model adjusted for correlations within patient and covariates including sex, age, marital status, sequence of cancer diagnosis, year of diagnosis, and Charlson comorbidity index.** $p < 0.05$

Cancers **2022**, *14*, 1296. https://doi.org/10.3390/cancers14051296
